# Statin Treatment in Hypercholesterolemic Men Does Not Attenuate Angiotensin II-Induced Venoconstriction

**DOI:** 10.1371/journal.pone.0103909

**Published:** 2014-09-29

**Authors:** Christoph Schindler, Kristina Guenther, Cosima Hermann, Carlos M. Ferrario, Christoph Schroeder, Sven Haufe, Jens Jordan, Wilhelm Kirch

**Affiliations:** 1 Clinical Research Center Hannover & Institute of Clinical Pharmacology, Hannover Medical School, Hannover, Germany; 2 Institute of Clinical Pharmacology, Medical Faculty, Technical University Dresden, Dresden, Germany; 3 Departments of Surgery, Internal Medicine – Nephrology, Physiology-Pharmacology; Wake Forest University School of Medicine, Winston-Salem, North Carolina, United States of America; Max-Delbrück Center for Molecular Medicine (MDC), Germany

## Abstract

Experimental studies suggested that statins attenuate vascular AT_1_ receptor responsiveness. Moreover, the augmented excessive pressor response to systemic angiotensin II infusions in hypercholesterolemic patients was normalized with statin treatment. In 12 hypercholesterolemic patients, we tested the hypothesis that statin treatment attenuates angiotensin II-mediated vasoconstriction in hand veins assessed by a linear variable differential transducer. Subjects ingested daily doses of either atorvastatin (40 mg) or positive control irbesartan (150 mg) for 30 days in a randomized and cross-over fashion. Ang II–induced venoconstriction at minute 4 averaged 59%±10% before and 28%±9% after irbesartan (mean ± SEM; P<0.05) compared to 65%±11% before and 73%±11% after 30 days of atorvastatin treatment. Plasma angiotensin levels increased significantly after irbesartan treatment (Ang II: 17±22 before vs 52±40 pg/mL after [p = 0.048]; Ang-(1–7): 18±10 before vs 37±14 pg/mL after [p = 0.002]) compared to atorvastatin treatment (Ang II: 9±4 vs 11±10 pg/mL [p = 0.40]; Ang-(1–7): 24±9 vs 32±8 pg/mL [p = 0.023]). Our study suggests that statin treatment does not elicit major changes in angiotensin II-mediated venoconstriction or in circulating angiotensin II levels whereas angiotensin-(1–7) levels increased modestly. The discrepancy between local vascular and systemic angiotensin II responses might suggest that statin treatment interferes with blood pressure buffering reflexes.

**Trial Registration:**

ClinicalTrials.gov NCT00154024

## Introduction

Among lipid lowering drugs, statins are particularly efficacious in ameliorating cardiovascular risk. While much of the improvement on statin treatment has been attributed to cholesterol reduction, additional “pleiotropic” actions may be involved. Experimental studies suggested that statins could modify renin angiotensin system responses through AT_1_ receptor downregulation [1]–[3] and altered vascular signalling including interference with Rho protein prenylation [4]. Few studies have assessed renin angiotensin system-modulating statin actions in man. The excessive pressor response to systemic angiotensin II infusion in hypercholesterolemic patients was normalized with statin treatment. Incremental norepinephrine elicited similar pressor responses regardless of cholesterol level and did not respond to statin treatment [5]. The authors suggested that pressor hypersensitivity in hypercholesterolemia is specific for angiotensin II likely through increased vascular responsiveness. However, systemic responses to vasoactive substances can be misleading because they are modified by baroreflex-mediated neurohumoral adjustments [6]. Indeed, statin treatment in hypercholesterolemic patients augmented rather than reduced forearm vasoconstriction to angiotensin II locally infused into the brachial artery [5]. Similarly, we reported previously that statins did not attenuate angiotensin II-induced venoconstriction in normocholesterolemic men. However, comparing venous responses to angiotensin II in healthy subjects and hypercholesterolemic patients revealed increased venoconstriction in hypercholesterolemic patients confirming the adequacy of the hand vein model to study this question [7]. We hypothesized that statin-induced downregulation of angiotensin II mediated venoconstriction may be unmasked in hypercholesterolemic subjects. Furthermore, we reasoned that statin-associated reduction in vascular angiotensin II responsiveness should lead to counterregulatory changes in angiotensin peptide release much like systemic AT_1_-receptor blockade. Given the known interaction between renin angiotensin and nitric oxide systems, we also tested for changes in endothelium dependent venodilation with statin treatment.

## Methods

The protocol for this trial and supporting CONSORT checklist are available as supporting information; see Checklist S1 and Protocol S1.

### Subjects

Between January and October 2006, we included 12 hypercholesterolemic men in our study. All men were otherwise healthy, as determined by history, physical exam, electrocardiogram, routine laboratory tests, complete blood count, and urine analysis. Exclusion criteria included a history of any significant disease, drug abuse, alcoholism, or active smoking. For allocation of the participants to the treatment arm, a computer-generated list of random numbers was used. Block size randomization was used. To implement the random allocation sequence as sequentially numbered containers were used. The random allocation sequence was generated by an independent biostatistician in the department of biostatistics and directly sent to the hospital pharmacy. Investigator and study personnel were blinded and not aware of the study medication. The study was approved by the Ethics Committee of the Medical Faculty of the Technical University, Dresden, Germany (EK 149112002). All subjects gave written informed consent before participating in the study. The trial was registered at ClinicalTrials.gov: NCT00154024.

### Study Design

**Figure 1:**
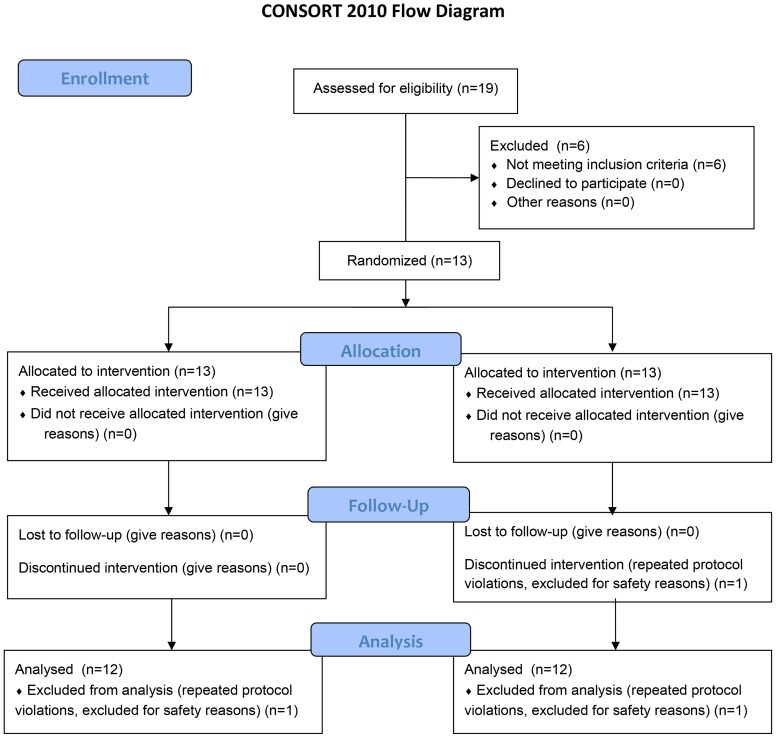
Consort Flow Diagram.

We conducted a 12-weeks randomized, double-blind, and crossover study (Figure 1). Subjects were randomized to ingest either 40 mg atorvastatin or 150 mg irbesartan daily for 30 days. Then, following an at least 30 days washout period, subjects who were first treated with atorvastatin were treated with irbesartan and subjects who first received irbesartan were treated with atorvastatin. Before and after each treatment period, we assessed venous responses using the dorsal hand vein compliance method. Furthermore, we obtained venous blood samples from an antecubital venous catheter for measurements of lipid levels, angiotensin peptides, and drug concentrations to check for compliance. Blood samples for angiotensin peptide measurements were collected in a protease inhibitor cocktail (25 mmol/L EDTA, 0.44 mmol/L o-phenantroline, and 0.12 mmol/L pepstatin A). We assessed supine blood pressure on the left arm following an at least five minutes resting phase using an oscillometric cuff (Dinamap, Critikon, Tampa, Fl). Serum atorvastatin and irbesartan concentrations served as compliance control.

## Venous Responses

We conducted all venous measurements in the morning hours with subjects in the supine position. Subjects had a light breakfast on study days. They were asked to refrain from caffeine-containing beverages for at least 12 hours before the investigations were begun.

The linear variable differential transducer (LVDT) technique is a useful approach to assess the influences of drugs in peripheral veins [7]–[9]. We inserted a 25-gauge needle in a suited dorsal hand vein. Then, we placed the LVDT probe on the same vein proximal to the puncture site and applied a brachial blood pressure cuff. Following instrumentation, we infused normal saline for 45 minutes. Then, we infused a constant angiotensin II dose for 24 minutes. Based on an earlier study [9] we applied 50 ng/min angiotensin II because this dose induces a reproducible constrictor response with negligible systemic blood pressure or heart rate changes. During angiotensin II infusion, we obtained LVDT measurements every 3 minutes by inflating the brachial cuff to 40 mm Hg for 1 minute. Following an at least 20 minutes washout period, we infused incremental phenylephrine doses (47–1500 ng/min) until vein diameter had stably decreased to 20% of the baseline value. On top of the continuous phenylephrine infusion, we infused incremental histamine doses (2-4-8-16-32-64-128 ng/min) together with the dose of phenylephrine constricting the vein to 20% basal vein size (ED_80_) for 10 minutes each. This enabled us to construct dose response curves. We obtained LVDT measurements in the last three minutes of each infusion step.

### Drugs

All intravenously applied drugs were diluted in normal saline solution. We used the following drugs: Phenylephrine hydrochloride (American Regent Laboratories, Shirley, NY), Angiotensin II (Merck Biosciences, Laeufelfingen, Switzerland). Histamine diphosphate was purchased from Sigma Chemicals (Munich, Germany). A sterile histamine solution for intravenous use was manufactured by the pharmacy of the university hospital.

## Analytical Methods

We measured venous plasma angiotensin II and angiotensin-(1–7) concentrations as described previously [7]. Briefly, samples were extracted in the presence of radiolabeled peptide; recovery of radiolabeled peptide averaged >65%, and results were corrected for recovery. The sample was reconstituted in assay buffer. Angiotensin II was measured using a radioimmunoassay (Alpco kit, Alpco, Windam, NH). Angiotensin-(1–7) was detected using a previously characterized antibody. Lower levels of detection were 0.8 pg per tube for Ang II and 2.5 pg per tube for angiotensin-(1–7). The intra- and interassay coefficients of variation were 12% and 22% for angiotensin II and 8% and 20% for angiotensin-(1–7), respectively. Determination of Serum Levels of Irbesartan and Atorvastatin (Day 20; Visits 3 and 7) Serum levels of atorvastatin and irbesartan were determined at day 20 of each treatment interval as a compliance control. Sample preparation was performed using solid-phase extraction (SPE). Atorvastatin and irbesartan concentrations were measured with liquid chromatography/tandem mass spectrometry (LC/MS/MS) working in the multiple-reaction monitoring mode (MRM) with specific transitions between m/z 559.1 (parent ion) and m/z 439.6 for atorvastatin and m/z 429.1 (parent ion) and m/z 206.7 for irbesartan using external calibration.

## Sample Size Calculation and Statistical Analysis

Based on data of intra- and inter-individual differences in hand vein responses of healthy subjects from previous studies [7], we calculated that inclusion of twelve subjects in a crossover study would provide a power of 80% detecting ≥12% changes in basal vein size. Results are expressed as mean ± standard error of the mean (SEM) unless otherwise stated. We tested normal distribution of our data with the Kolmogorov-Smirnov test. To test for differences at single time points during the 24-minutes measurement period we used a two-way analysis of variance (ANOVA) with *Bonferroni's post hoc* tests. Finally we analyzed a possible interaction between treatments (atorvastatin versus irbesartan) and time (before versus after treatment) on the response of hand vein constriction with a two-way ANOVA for repeated measures. Changes in angiotensin peptide concentration from venous blood samples before and after each treatment period were analysed with *Students T-Test* for paired samples. For all calculations, the statistical software package SPSS Version 20 (IBM, Armonk, NY) was used.

## Results

**Figure 2:**
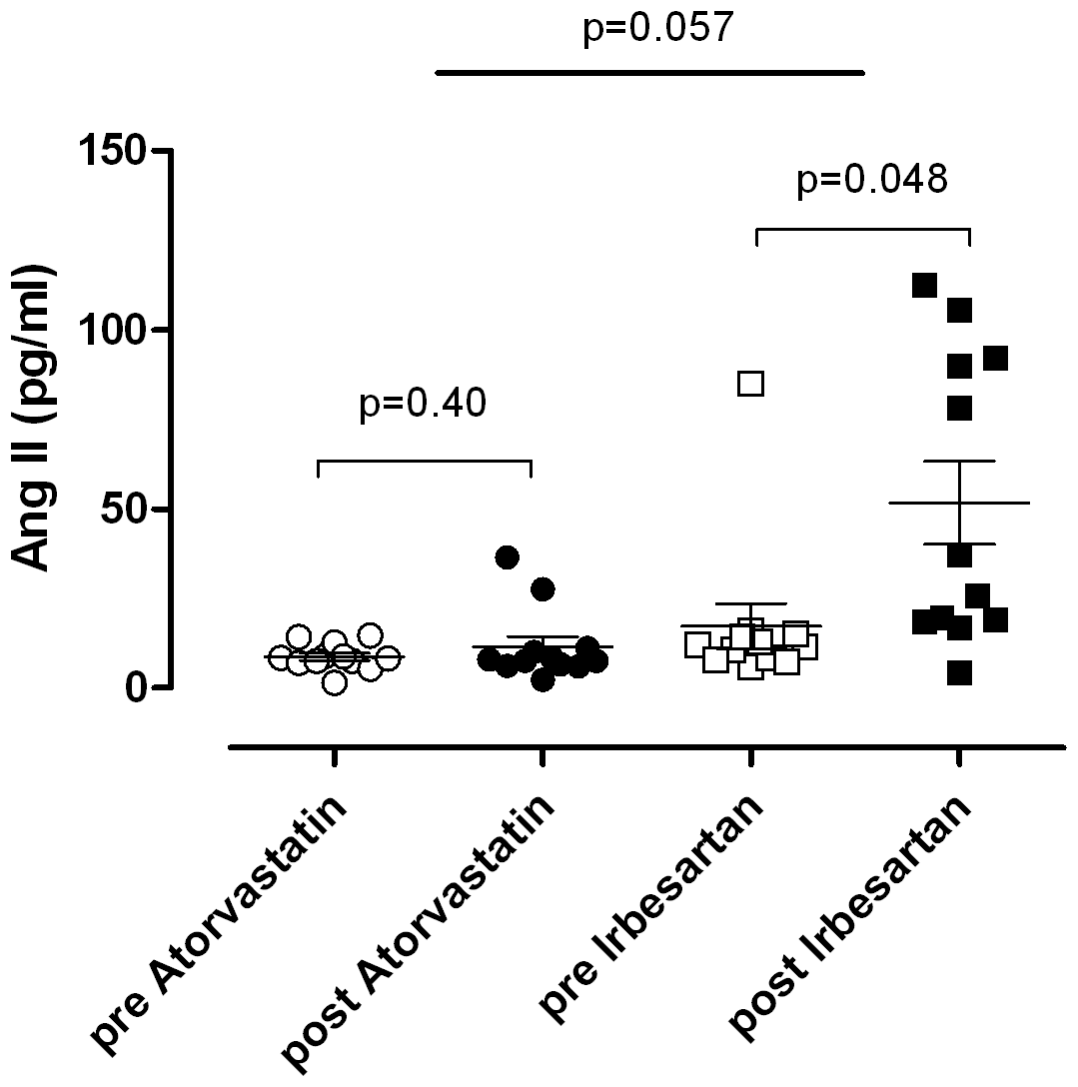
Atorvastatin and irbesartan influences on plasma concentrations of angiotensin II. Within-group differences were analyzed with *Students* paired t-test and between-group differences with a two-way ANOVA. Data are expressed as individual values and as mean ± SEM.

**Figure 3:**
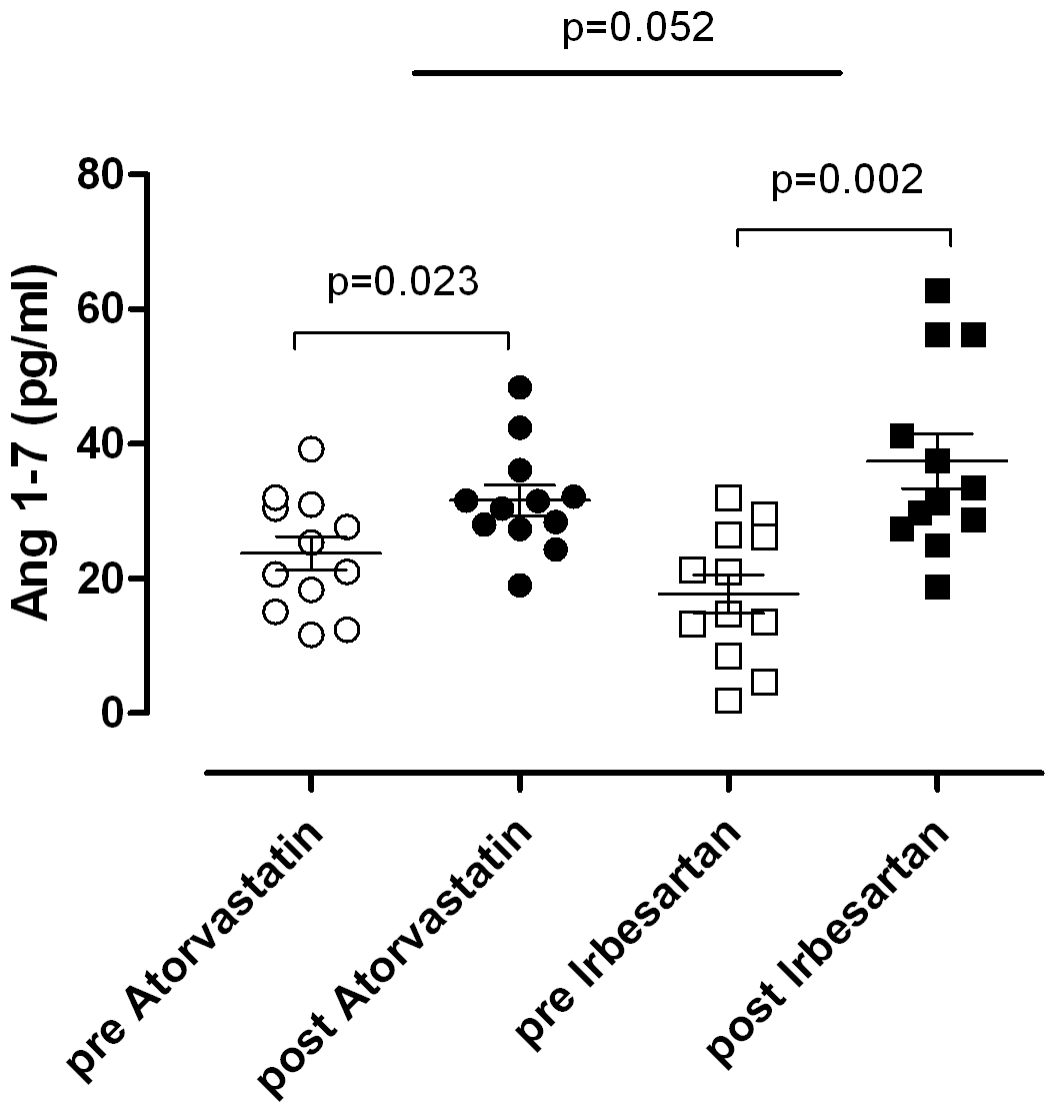
Atorvastatin and irbesartan influences on plasma concentrations of angiotensin-(1–7). Within-group differences were analyzed with *Students* paired t-test and between-group differences with a two-way ANOVA. Data are expressed as individual values and as mean ± SEM.

19 subjects were initially enrolled for elegibility. 6 subjects did not fulfil all inclusion criteria and were excluded. 13 patients were randomized of which one subject was excluded again due to non-compliance and protocol violation. The other 12 hypercholesterolemic men (age 33±7 years, body mass index 25±2 kg/m^2^, total cholesterol 6.6±1.1 mmol/l; LDL cholesterol: 4.7±1.1 mmol/l; HDL cholesterol: 1.6±0.2 mmol/l) were studied and analyzed according to the protocol. All subjects tolerated treatment with atorvastatin and irbesartan equally well and reported no serious adverse events. On treatment, sufficient atorvastatin and irbesartan plasma concentrations were detectable in all subjects. Mean plasma concentration on treatment day 30 was 17±13 ng/mL (range 3–39 ng/mL) for atorvastatin and 3590±1230 ng/mL (range 1845–5777 ng/mL) for irbesartan. Plasma total cholesterol was 6.5±1.3 mmol/L before and 3.9±0.9 mmol/L after atorvastatin treatment (p<0.001) and 6.4±1.2 mmol/L before and 6.2±1.2 mmol/L after irbesartan treatment (p = 0.178). Low density cholesterol was 4.8±1.4 mmol/L before and 2.3±0.9 mmol/L after atorvastatin treatment (p<0.001) and 4.6±1.3 mmol/L before and 4.5±1.2 mmol/L after irbesartan treatment (p = 0.351). High density cholesterol did not change with either intervention. The results of angiotensin II and angiotensin-(1–7) measurements are given in Figures 2 and Figure 3. While angiotensin II did not change with atorvastatin, we observed a robust increase with irbesartan. Angiotensin-(1–7) plasma concentrations increased with both interventions.

**Figure 4:**
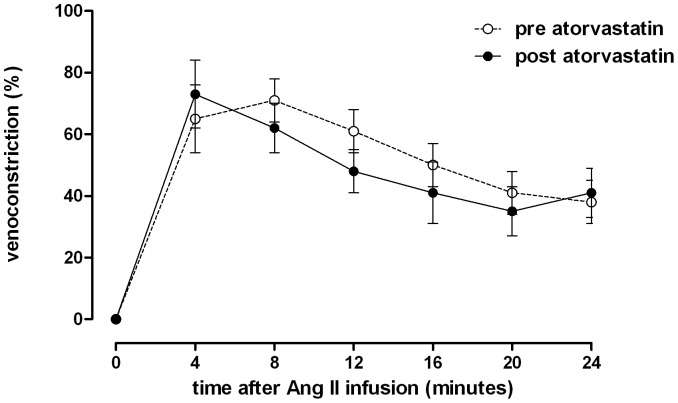
Time course of dorsal hand vein constriction with constant angiotensin II infusion. Infusion rate: 50 ng/min over 24 minutes before (pre) and after (post) treatment with atorvastatin. Differences between pre- and post-treatment were analyzed with a two-way ANOVA with *Bonferroni's post hoc* tests to test for differences at single time points. Data are expressed as mean ± SEM.

**Figure 5:**
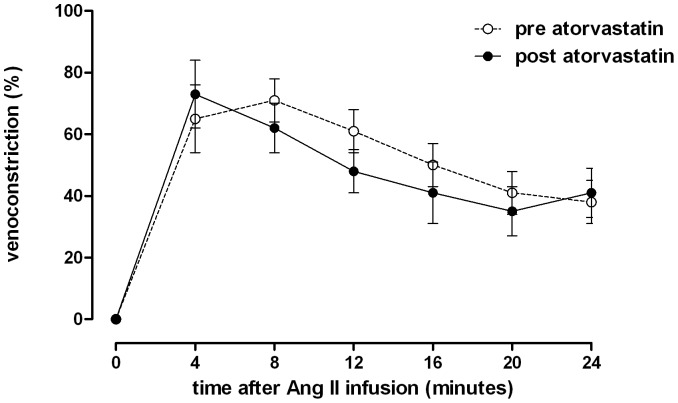
Time course of dorsal hand vein constriction with constant angiotensin II infusion. Infusion rate: 50 ng/min over 24 minutes before (pre) and after (post) treatment with irbesartan. Differences between pre- and post-treatment were analyzed with a two-way ANOVA with *Bonferroni's post hoc* tests to test for differences at single time points. Data are expressed as mean ± SEM.

Venous responses to local angiotensin II infusion before and after atorvastatin and before and after irbesartan treatment are illustrated in Figure 4 and 5. Angiotensin II infusion elicited rapid venoconstriction peaking between 4 and 8 minutes after commencement of the infusion. Thereafter, the response gradually decreased during the remainder of the infusion period. Atorvastatin treatment did not significantly alter angiotensin II-induced venoconstriction at any time point during angiotensin II infusion. Acute venoconstriction (4 minutes after start of infusion) was 59±10% before and 28±9% after irbesartan treatment (p<0.05). In contrast venoconstriction was similar with 65±11% before and 73±11% after atorvastatin treatment.

**Figure 6:**
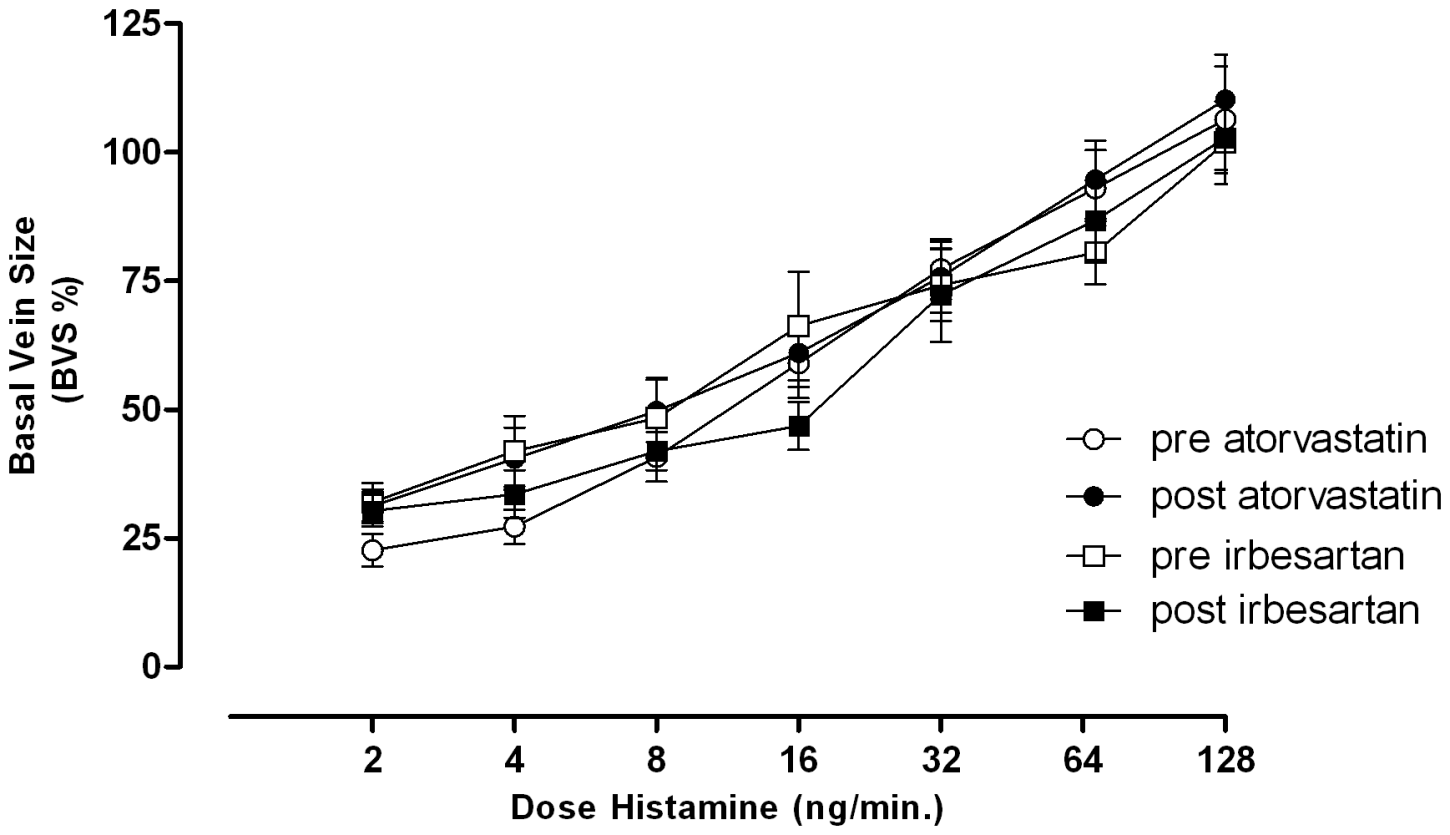
Dilation of preconstricted dorsal hand veins elicited by incremental histamine infusions. Between-group differences were analyzed with a two-way ANOVA. Data are expressed as mean ± SEM.

The phenylephrine dose constricting the vein to approximately 20% of the basal diameter was 819±313 ng/min before and 992±358 ng/min after atorvastatin (p>0.05) compared with 819±314 ng/min before and 807±306 ng/min after irbesartan treatment (p>0.05). Histamine-induced endothelium-dependent venodilation in phenylephrine pre-constricted veins did not change with either atorvastatin or irbesartan treatment (Figure 6).

Blood pressure and heart rate did not significantly change during local drug infusions except during angiotensin II-infusion which slightly but significantly increased systolic BP from 115±10 to 120±10 mm Hg and diastolic BP from 71±7 to 74±9 mm Hg (p<0.05) compared to normal saline infusion.

## Discussion

The important finding of our study is that statin treatment did not elicit statistically significant reductions in angiotensin II-induced venoconstriction in normotensive hypercholesterolemic men as hypothesized. However, Ang II-induced venoconstriction was non statistically significant reduced in the present study with hypercholesterolemic men whereas results in our earlier study in normocholesterolemic men showed increased venoconstriction after statin treatment [7]. Furthermore, statin treatment did not increase systemic angiotensin II concentrations, which is the expected response when vascular angiotensin II responsiveness is decreased. In contrast, AT_1_ receptor blockade with irbesartan substantially attenuated the venoconstrictor response to angiotensin II while profoundly raising circulating angiotensin II concentrations. Both, statin treatment and AT_1_ receptor blockade were associated with small but significant increases in plasma angiotensin-(1–7)-levels. Possibly, atorvastatin augmented angiotensin II to angiotensin-(1–7) conversion by angiotensin converting enzyme 2 (ACE 2) as previously shown experimentally [10]. ACE 2 attenuates atherosclerotic lesions by targeting vascular cells [11]. Together with earlier investigations testing local vascular responses to angiotensin II in human subjects [1], our study challenges the idea that statins elicit a substantial part of their beneficial effect through circular renin angiotensin system inhibition.

Both, resistance vessels and venous capacitance vessels contribute to the pathogenesis of arterial hypertension. Indeed, decreased venous capacitance has been described in several hypertensive animal models [12], [13]. Yet, in human subjects, neither resistance vessels in the forearm [5], [14], [15] nor peripheral veins [7] showed reductions in angiotensin II-mediated vasoconstriction with statin treatment. These observations are at variance with previous studies in arterial vessels of animals [16], [17] suggesting AT_1_-receptor-downregulation through statin treatment [16], [18]. The differential findings may be related in part to their higher sympathetic control of venous tone in humans compared to rodents since the erect posture is a human characteristic.

Our methodology was suitable to show reductions in vascular angiotensin II responsiveness as AT_1_ receptor blockade at a modest dose potently inhibited the response. Similar inhibition of angiotensin II mediated venoconstriction in hand veins has been previously observed [7], [8]. Measurements of drug concentrations on treatment confirmed that all subjects had ingested the study medication. Furthermore, we observed more than 50% reductions in plasma low density lipoprotein concentrations consistent with sufficient statin dosing. A previous study showed reduced vasoconstriction in isolated aortic rings of normocholesterolemic spontaneously hypertensive rats following the 4-weeks statin treatment period [16]. The investigators proposed that AT_1_-receptor down-regulation and reduced oxidative stress may have contributed to the response. Yet, animals were treated with extraordinary high statin doses (50 mg/kg body weight) such that these findings cannot simply be extrapolated to the clinic. Finally, species differences could affect the vascular response to statin treatment.

Strong interactions between vascular angiotensin II and nitric oxide responses have been observed previously [19], [20]. We, therefore, assessed endothelium dependent venodilation. Because of a striking variability in acetylcholine-mediated hand vein dilation [21], we applied the endothelium-dependent vasodilator histamine [22]. Histamine-induced venodilation is highly reproducible and reversed through nitric oxide-synthase inhibition [23]. Endothelium dependent venodilation of pre-constricted hand veins was not altered with statin treatment. The observations suggest that opposing changes in endothelial function did not mask a change in angiotensin II responsiveness.

Our results on plasma angiotensin II and angiotensin-(1–7) concentrations extend results obtained in hand veins. As expected [7], AT_1_ receptor blockade with irbesartan led to robust increases in circulating angiotensin II concentrations. In contrast, plasma angiotensin II levels did not change in response to statin treatment. The observation suggests that statin treatment may not cause decreased vascular angiotensin II responsiveness such that a counter regulatory response did not occur. An alternative, less likely, explanation is that statin treatment attenuated vascular angiotensin II responsiveness and angiotensin II release to a similar degree.

Both, statin treatment and AT_1_ receptor blockade modestly increased circulating angiotensin-(1–7) concentrations. In our previous study in normocholesterolemic subjects, angiotensin-(1–7) was unchanged with statin treatment, suggesting that basal cholesterol levels may modulate the response. These findings could be clinically relevant because angiotensin-(1–7) opposes angiotensin II actions through release of bradykinin, vasodilator prostaglandins, and endothelial nitric oxide. Apparently, vasodilator effects of Ang-(1–7) are mediated by the G-protein-coupled receptor mas [24]–[26]. Remarkably, treatment with selective Ang-(1–7) antibodies or the Ang-(1–7) antagonist –D-[Ala7]-Ang-(1–7) attenuates the antihypertensive response to angiotensin converting enzyme or AT_1_-receptor inhibition in animals [27], [28]. Our results point towards a potential contribution of Ang-(1–7) to pleiotropic responses to statin treatment in hypercholesterolemic patients.

## Perspective

In contrast to experimental studies in animals in the arterial vascular bed, we and others observed no significant reduction in angiotensin II-induced venoconstriction in human subjects with or without hypercholesterolemia. It is difficult to reconcile these observations with an earlier study suggesting that systemic angiotensin II infusion elicits a greater response in patients with elevated cholesterol levels and that statin treatment ameliorates the response. Systemic application of vasoactive agents is indispensable in testing the overall responsiveness of the cardiovascular system. However, systemic responses to vasopressor agents are affected by, both, vascular sensitivity and counter regulatory adjustments through baroreflex buffering mechanisms [6]. Indeed, systemic angiotensin II infusion elicits baroreflex-mediated reductions in heart rate and sympathetic vasomotor tone in human subjects [29]. The mechanism restrains the pressor response to systemic angiotensin II infusion. We speculate that an improvement in baroreflex sensitivity rather than changes in vascular sensitivity on statin treatment attenuates the pressor response to systemic angiotensin II infusion. Indeed, statin treatment increased baroreflex sensitivity through actions on afferent [30] and efferent [31] baroreflex pathways in rats. Furthermore, AT_1_ receptors are expressed at multiple sites in the central nervous system governing cardiovascular reflex responses [32]. Statin-induced changes in central nervous AT_1_ receptor signalling could conceivably alter the response to systemic angiotensin II infusion. Overall, our study illustrates the difficulty of translating some of the exciting findings in renin angiotensin system biology into the clinic.

## Novelty and Significance

We observed responses to vasoactive drugs in veins. Our results can not necessarily be extrapolated to the arterial vascular system. Comparing native venous responses to angiotensin II in healthy subjects from our former study [7] with hypercholesterolemic patients in our present study reveals increased venous responsiveness in hypercholesterolemic patients compared to healthy subjects confirming the adequacy of the hand vein model to study this question. A previous study suggested that hypercholesterolemic patients are hypersensitive to systemic angiotensin infusions. Systemic responses to angiotensin II are confounded by neurohumoral reflex adjustments. In hypercholesterolemic men, we tested the local hand vein response to angiotensin II before and after treatment with a statin or with an AT_1_ receptor-antagonist and measured angiotensin II and angiotensin-(1–7) plasma levels.

## Summary

In our study, statin treatment in hypercholesterolemic men did not blunt venous constriction elicited by angiotensin II as suggested by in vitro results whereas AT_1_ receptor blockade profoundly attenuated the response. We show for the first time that atorvastatin treatment significantly increases plasma levels of the pleiotropic RAS-peptide Angiotensin-(1–7) in hypercholesterolemic patients whereas atorvastatin treatment of healthy subjects does not affect this peptide. Our observation of increased Angiotensin-(1–7)-levels suggests that RAS-inhibitory effects, might contribute to the beneficial vascular effects of atorvastatin summarized as pleiotropic effects. The discrepancy between local vascular and systemic angiotensin II responses might suggest that statin treatment interferes with blood pressure buffering reflexes.

## Supporting Information

Checklist S1 **CONSORT Checklist.**
(DOC)

Protocol S1 **Trial Protocol.**
(DOCX)
