# Correction: Statin Treatment in Hypercholesterolemic Men Does Not Attenuate Angiotensin II-Induced Venoconstriction

**DOI:** 10.1371/journal.pone.0112205

**Published:** 2014-10-23

**Authors:** The PLOS ONE Staff

Figure 5 is a duplicate of Figure 4. The authors have provided the correct Figure 5 here.

**Figure 5:**
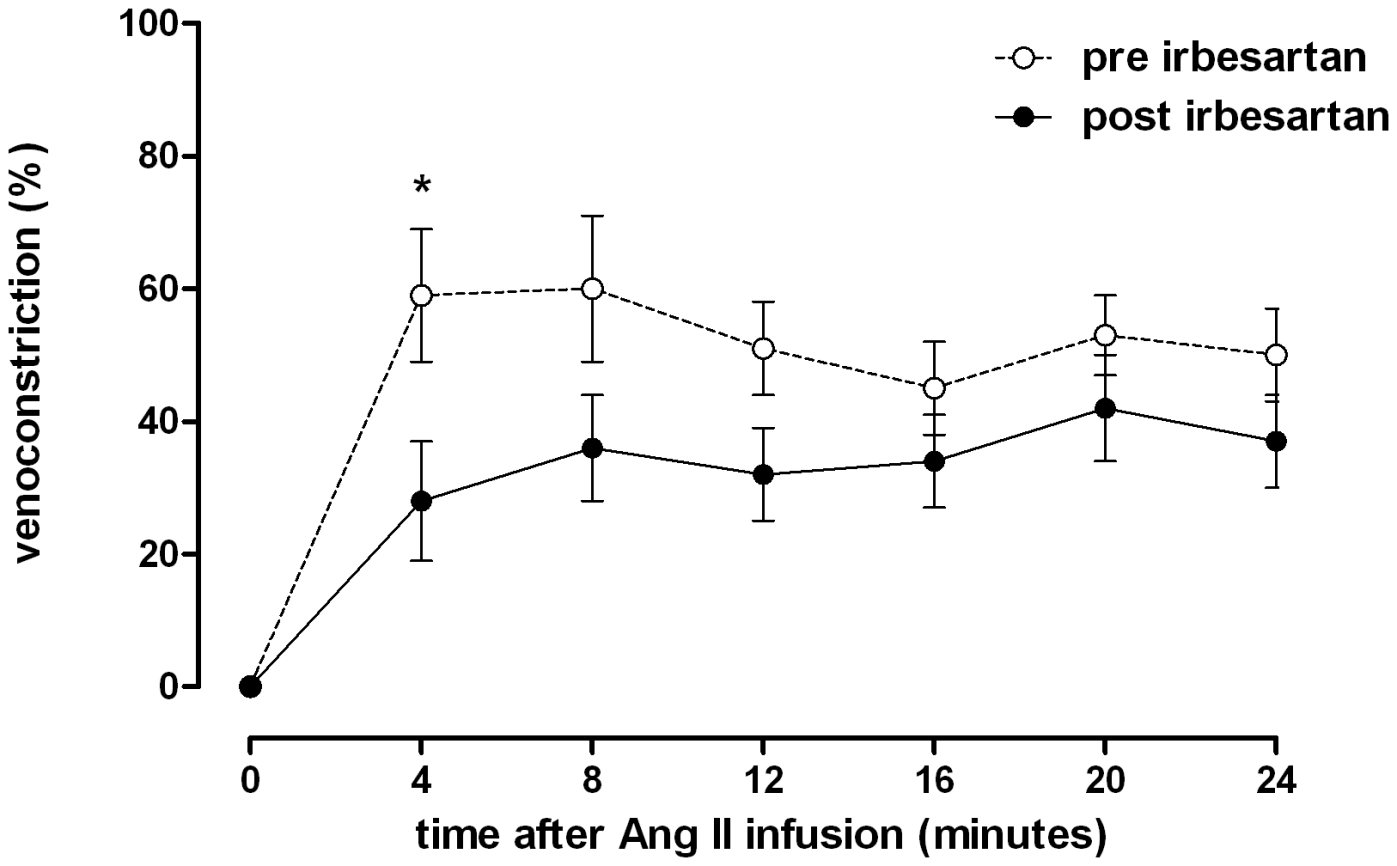
Time course of dorsal hand vein constriction with constant angiotensin II infusion. Infusion rate: 50 ng/min over 24 minutes before (pre) and after (post) treatment with irbesartan. Differences between pre- and post-treatment were analyzed with a two-way ANOVA with *Bonferroni's post hoc* tests to test for differences at single time points. Data are expressed as mean ± SEM.
