# Supplementary material for: Statin Treatment in Hypercholesterolemic Men Does Not Attenuate Angiotensin II-Induced Venoconstriction
Source: PLoS One. 2014 Sep 29;9(9):e103909. doi: 10.1371/journal.pone.0103909 (PMC4179232; doi:10.1371/journal.pone.0103909)
Supplement: Protocol S1 — Trial Protocol. (DOCX) [file pone.0103909.s002.docx]

**Protocol – Clinical Trial number IKPD 03-02**

**Amendment: hypercholesterolemic patients**

(abbreviated english translation, original protocol in german language)

**Title of protocol:** Comparison of local vascular effects of angiotensin II and histamine before and after oral drug therapy with irbesartan and atorvastatin in hypercholesterolemic patients.

**Phase:** no phase – experimental clinical pharmacological trial

**Aim of study:** Comparison of angiotensin II - induced venoconstriction and histamine-induced venodilation before and after oral drug therapy with irbesartan and atorvastatin.

**Study Design:** monocentric, double blind, randomized, crossover-study

**Study Centre:** Institute of Clinical Pharmacology, Medical Faculty, Technical University Dresden; Fiedlerstrasse 27, D – 01307 Dresden, Germany

**Subjects:** n = 12 hypercholesterolemic subjects

**Study Duration:** 12 – 18 months

**Dosing, drug application:** oral ingestion of 40 mg atorvastatin and 150 mg irbesartan in a double blind randomized manner crossover manner for 30 days each, washout between oral drug therapy intervals at least 4 weeks. Preconstriction of dorsal hand vein with incremental phenylephrine doses (47–1500 ng/min) until vein diameter had stably decreased to 20% of the baseline value. Intravenous stimulation with angiotensin II mini dose (50 ng/min.); construction of dose response curve with incremental histamine doses (2-4-8-16-32-64-128 ng/min).

**Inclusion criteria:** Male hypercholesterolemic patients aged between 18 and 45 years, non-smokers, after oral and written informed consent.

**Exclusion criteria:** Any relevant changes according to the discretion of the investigator in physical examination, ECG, clinical chemistry, hematology, severe allergic diathesis; body weight > 15% different from normal according to Broca.

**Primary endpoint:** % Change in dorsal hand vein diameter.

**Safety parameter:** routine laboratory at screening day; continuous monitoring of blood pressure, heart rate and ECG during experimental examination with phenylephrine, angiotensin II and histamine.
